# Testing the psychometric properties of the risk-rescue rating scale: a lethality measure for suicide attempts

**DOI:** 10.1186/s13033-025-00662-0

**Published:** 2025-01-30

**Authors:** Tormod Stangeland, Ketil Hanssen-Bauer, Johan Siqveland

**Affiliations:** 1 Division of Mental Health Services, Akershus University Hospital, Lørenskog, Norway; 2 Campus Ahus, Institute of Clinical Medicine, University of Oslo, Oslo, Norway; 3 National Centre for Suicide Research and Prevention, Institute of Clinical Medicine, University of Oslo, Oslo, Norway

**Keywords:** Suicide, Lethality, Interrater reliability, Risk-rescue rating scale, Suicide intent scale

## Abstract

Health personnel lack a common standard for assessing lethality of suicide attempts. This may lead to inconsistent assessments and unclear reports about suicide attempts. We argue that the Risk-Rescue Rating Scale (RRRS) may help in resolving this problem. It is a measure based on observable indications of the medical danger of a suicide attempt and of the patient’s efforts to avoid or achieve rescue. The instrument is a clinician-rated supplement to self-reports and can be administered in a few minutes and learned in a single brief teaching session. We adapted the RRRS for contemporary use in a Norwegian acute adolescent mental health service clinic. We developed a training program for clinicians, a user manual, and a series of five video-based role-played interview cases for reliability testing. In this study, we recruited 28 clinicians with professional backgrounds typical of Norwegian mental health personnel. They rated five role-played video interviews using the RRRS and the well-established interview instrument the Suicide Intent Scale (SIS) and obtained 140 sets of scores. We estimated the interrater reliability (intraclass correlation coefficient [ICC]) to be .93 for the RRRS and .94 for the SIS, both excellent levels. Correlation was .80 between the RRRS and SIS items that were similar to the RRRS and .53 for SIS items measuring other topics, indicating good concurrent and discriminant validity. Adopting a common standard for communicating about suicide attempts can improve clinical practice, and the RRRS may prove to be a reliable and practical candidate for this task.

*Public significance statement:* This study evaluated the Risk-Rescue Rating Scale for use by clinicians in assessing the lethality of suicide attempts. Our findings indicate that this instrument has excellent reliability and can help clinicians better understand and communicate about suicide attempts, ultimately leading to more effective interventions and improved care for individuals at risk of suicide.

A suicide attempt is typically defined as “a nonfatal self-directed potentially injurious behavior with any intent to die as a result of the behavior. A suicide attempt may or may not result in injury” [4], p. 21). According to this broad definition, someone attempting to end their own life by consuming a box of vitamin C pills is regarded as having engaged in a suicide attempt, even if the act poses very little medical threat. The broad definition of suicide attempts alerts clinicians to suicide attempts of low lethality, which is clearly beneficial. Raising awareness among clinicians can improve identification and treatment.

However, for clinicians tasked with making decisions about safety measures following a suicide attempt, it is crucial to assess the risk of further suicidal behavior. Hospitalization and other invasive interventions may be relevant, especially if clinicians view the suicide attempt as dangerous and if there is a high chance of repeat attempts. The broad definition of suicide attempts is not helpful for distinguishing between the various levels of danger. Lacking a common standard, clinicians may interpret and weigh factors differently and may arrive at different reports and recommendations for treatment, in turn impairing communication within the health services. More consistent information gathering, assessment, and communication about suicide attempts may improve clinical practice. Also, when assessing patients with a history of multiple suicide attempts, clinicians would benefit from nuanced knowledge of previous suicide attempts to ascertain trends toward more dangerous attempts and habituation to suicidal behavior [33]. Finally, patients who survive suicide attempts of different degrees of lethality may be driven by different motivations and may benefit from different kinds of therapeutic intervention [32]. Differentiating between suicide attempts might reveal patterns of motivation and capability, instead of treating it as a uniform concept.

The term “lethality” refers to the seriousness or level of danger associated with suicide attempts, potentially providing the basis for a common standard of assessment. Several overlapping definitions are in use, including definitions of serious suicide attempts [16] Central elements of lethality include the medical severity of the attempt, the degree of preparation, the risk of fatality, and the probability of rescue by others. Suicidal intent, defined as the desire and expectancy of a fatal outcome, is not part of lethality. Lethality is an important predictor for later suicidal behavior. The lethality or method used in a suicide attempt has been found to predict the lethality and risk of later suicide attempts [28] and eventual suicide death [26, 29].

Established lethality instruments include the Suicide Intent Scale (SIS) [2], the Lethality of Suicide Attempt Rating Scales [3, 30], the Self-Inflicted Injury Severity Form [25] and the Risk-Rescue Rating Scale (RRRS) [35]. These instruments were all developed several decades ago, and while they continue to see some use, there is little current research and development in the lethality measurement field.

In contemporary suicide research, items relevant for measuring lethality are part of the comprehensive assessment tool Self-Injurious Thoughts and Behavior Interview (SITBI) [19]. Also, the Columbia Suicide Severity Rating Scale [24], a clinical interview for risk assessment, covers a range of suicidal thoughts and behaviors and collects information about suicide attempts, such as preparation, interruption, and the severity of medical injury, similar to that required by the RRRS. However, the interview format of both of these instruments is based mainly on self-reported information from the patient and less on medical information. Also, the clinical assessment of likelihood of a fatal outcome without clear criteria for the assessment introduces problems for reliability.

The RRRS is noteworthy for its emphasis on high reliability, its reliance on observable rating criteria with minimal need for subjective interpretation, and its provision of a concise yet nuanced measure of lethality, including actions taken to avoid or achieve rescue. It includes medical information and does not depend on patient self-reports, which are not always available or reliable in acute settings. Spirito et al. [31] examined the RRRS used with adolescents and recommended more specific rating criteria. However, to our knowledge, no such study has been published. In recent years, the RRRS has occasionally been used as an outcome measure of the severity of suicidal attempts [13, 21], but no recent studies have assessed its reliability and validity.

This paper reports on our study of the interrater reliability and concurrent validity of the RRRS. We translated the scale into Norwegian, prepared a rating manual, and developed a series of video interview cases. We compared the RRRS ratings with the corresponding SIS ratings.

The aim of the present study was to investigate four research questions in a setting where.

mental health service clinicians use the RRRS to rate the lethality of constructed suicide attempt cases:

1. What is the interrater reliability of their ratings?
2. Do they rate the cases at the expected level and range?
3. Do RRRS ratings differ between clinical groups with different professional roles?
4. Do RRRS ratings correlate as expected with SIS ratings?

## Methods

### Transparency and openness

The study design and analysis were not preregistered. All data, analysis codes, and research materials are available upon request from the first author.

### Participating raters and data collection procedure

*We chose to involve a larger group of participating clinicians with different roles and experience, since we wished to compare groups and ensure a more representative sample of raters than when the measure is used in clinical practice.* Our participating raters were clinicians recruited from a large Norwegian mental health clinic for adolescents, which offers acute and medium-length inpatient and ambulatory treatment. We recruited four groups of clinicians: (1) medical doctors and psychologists within the inpatient ward, (2) nurses and social workers within the inpatient ward, (3) an ambulatory team based at the mental health clinic, and 4) a liaison team offering mental health services to a somatic acute clinic for children and adolescents. The latter two teams consisted of experienced medical doctors, psychologists, and therapists from other occupational backgrounds, specialized in assessing suicidal adolescents. Taken together, these clinicians are responsible for assessing nearly all the adolescent suicide attempt cases in the catchment area that receive further help after emergency room examination.

We arranged a training program to enable the clinicians in these four groups to use the RRRS and SIS as routine measures. The participants received a half-hour instruction on the use of the test instruments. Afterwards, they watched a series of recorded role-played video cases and independently rated each case using both the RRRS and SIS. The participants had no prior experience with the instruments before participating in the study.

## Measurements

### The risk-rescue rating scale (RRRS)

The RRRS [35] makes use of clinician ratings based on observable rating criteria, which were chosen because of their minimal need for subjective interpretation. The development of the original test instrument yielded interrater reliability measures ranging from 0.93 to 0.95. It consists of five items constituting the Risk subscale, which assesses the medical danger of a suicide attempt and aspects included in several lethality instruments (eg [30]) and measures of serious suicide attempts [1]. The Rescue subscale addresses a less frequently assessed aspect, through five items assessing the patient’s actions to avoid or achieve rescue. Based on all available information from medical health records, the patient, and witness reports, the clinician rates the items on a three-point scale. The two subscales are combined in a short assessment of the event, for instance, “a moderate risk suicide attempt, with high chance of rescue”, a practical format for clinical communication. The subscale ratings can be aggregated into a numerical total rating, which is useful for research purposes, monitoring, and practice evaluation.

We developed a Norwegian version of the instrument (“Risk-Redning”), adapting the terms and rating criteria used in the original scale to the Norwegian context. The instructions and test items in the original RRRS are brief and mainly consist of single terms and concrete phrases. Translation decisions were made by consensus discussions among a group of experienced clinicians at our mental health clinic, with input from early experiences with practical testing. Translation of the brief instrument text was straightforward and mostly concerned with finding appropriate Norwegian terms and contemporary names for health services. Following suggestions from Spirito and colleagues (1991) on how to improve the interrater reliability of the RRRS, we prepared a user manual with specific rating instructions, including the use of wordings that reduced the impact of subjective judgment during the rating process.

### The suicide intent scale (SIS)

In order to study the concurrent validity of the RRRS, we compared it to the well-known Suicide Intent Scale (SIS) [2]. The SIS is a semistructured patient interview that assesses overlapping but not identical concepts to those comprising the RRRS. The first eight SIS items, constituting the Method subscale, assess the patient’s descriptions of the lethality of the suicide attempt [18], similar to the RRRS items. The latter seven SIS items, constituting the Intent subscale, assess the patient’s own descriptions of suicidal intent, which the RRRS explicitly excludes. A clinician rates all fifteen items on three-point scales, and the SIS Total is the sum of all items. Both the total and subscale ratings were included in our study to ascertain both similarities and differences in comparisons with the RRRS.

A major review [8] identified 14 studies on the interrater reliability of the SIS total score and reported reliability coefficients ranging from 0.74 to 0.95. The SIS has been translated into a Norwegian version, which has been used in several studies [9, 11, 12, 23].

### Test material: clinical video cases

We filmed five video cases of clinical interviews in which experienced clinicians role-played patients admitted to the clinic after a recent suicide attempt. Each interview lasted 15 to 20 min and covered the relevant information for rating the RRRS and SIS. Each case also included a short referral document summarizing the treatment and available health records at intake. We constructed the cases to (1) represent typical suicidal patients referred to the clinic, (2) describe the full range of medical severity and suicidal intent, and (3) serve as realistic portrayals of patients, not cases designed to fit easily into the rating definitions in the user manual. Six clinicians with experience with the patient group prepared and role-played the video cases and constructed the scenarios to fit our plan for the expected ratings. Table 1 contains a brief description of the video cases and our expected rating levels for four measures: the RRRS Total, the Risk subscale, the Rescue subscale, and SIS Total.

**Table 1:** Expected rating levels and ranges for five video cases, for RRRS Total and subscales, and SIS total

	RRRS Total (range 16–83)	Risk subscale (range 5–15)	Rescue subscale (range 5–15)	SIS Total (range 0–30)
Case 1 hanging near home expected rating range	Moderate (40–50)	Moderate (9–10)	Moderate-High (12–13)	Moderate (7–17)
Case 2 corrosive ingestion expected rating range	High (70–83)	High (13–15)	Low (5–7)	High (18–30)
Case 3 impulsive strangulation expected rating range	Moderate (50–60)	Moderate (9–10)	Low (5–7)	Moderate (7–17)
Case 4 cutting near helpers expected rating range	Low (16–30)	Low (5–6)	High (14–15)	Low (0–6)
Case 5 running on railways expected rating range	Moderate (30–40)	Low-Moderate (7–8)	Moderate (10–11)	Moderate (7–17)

*RRRS* The risk-rescue rating scale, *SIS* suicide intent scale

### Statistical analyses

We conducted the following statistical analyses: central tendency measures for each case and both instruments, correlations between the subscales and total ratings, intraclass correlation coefficient (ICC) estimates for the total ratings and individual items for both instruments, and ANOVA testing to assess the influence of clinical team grouping on the RRRS ratings for each case. To ensure a reliable ICC analysis, we employed imputation techniques, replacing missing responses with the most frequently occurring rating for the relevant case.

We used Koo and Li’s guidelines for interpreting ICCs [15]: Values less than 0.5 are indicative of poor reliability, values between 0.5 and 0.75 indicate moderate reliability, values between 0.75 and 0.9 indicate good reliability, and values greater than 0.90 indicate excellent reliability. The data analysis was performed using version 29 of the SPSS statistical package.

### Ethics

The RRRS and SIS rating sheets were anonymous. Participants who wished to contribute to the study submitted their rating sheets after completion. All workshop participants chose to do so. The rating sheets contained no personal information, except for the participants’ clinical role. Since anonymous professional participants voluntarily submitted rating sheets containing no sensitive information, we concluded that approval from the Data Protection Officer was not necessary. The present study is part of a larger project approved by the Norwegian Regional Ethics Committee (REK approval number 322341).

## Results

### Participating raters

Fifty clinicians attended one or more training sessions and rated one or more cases. Since our analysis required complete data sets, our study included only the 28 participants who rated all five cases. Twenty-one participants were female, and seven were male. This reflects the national distribution of health personnel in acute adolescent mental health clinics in Norway. The ratings from the group with complete data sets were almost identical to those from the group with incomplete data sets. No differences were near statistically significant levels, according to t tests comparing the two group means for each case.

Twenty-eight clinicians, each rating five cases, contributed a total of 140 case ratings, each encompassing both RRRS and SIS assessments. The RRRS had no missing response items. The 140 SIS case ratings contained 2,100 SIS response items, of which we found 15 missing response items, a missing rate of 0.71%. We considered them to be missing completely at random [34], at a rate well within acceptable limits for missing data. We imputed the most frequent rating of the item of the relevant case for the 15 missing responses.

### Interrater reliability of the RRRS and SIS ratings

For our primary research question, we sought to examine interrater reliability in our study. In Table 2, we present ICC estimates with 95% confidence intervals for the RRRS Total and its subscales across all five cases. In Table 3, we present the corresponding estimates for the SIS Total and its subscales.

**Table 2:** ICC for the RRRS total and subscale ratings

	ICC	95% Confidence Interval		F Test with True Value 0			
		Lower Bound	Upper Bound	Value	df1	df2	Sig.
RRRS total	0.93	0.82	0.99	355.92	4	108	< 0.001
Risk subscale	0.87	0.70	0.98	212.42	4	108	<0 .001
Rescue subscale	0.94	0.84	0.99	372.20	4	108	<0 .001

N = 140. Two-way random effects model where both rater effects and measures effects are random, single measures. Type A ICC using an absolute agreement definition

*ICC* Intraclass correlation coefficient, *RRRS* the risk-rescue rating scale

**Table 3:** ICC for the SIS total and subscale ratings

	ICC	95% Confidence Interval		F Test with True Value 0			
		Lower bound	Upper bound	Value	df1	df2	Sig.
SIS Total	0.94	0.83	0.99	394.10	4	108	<0 .001
Method subscale	0.85	0.67	0.98	157.44	4	108	<0 .001
Intent subscale	0.94	0.83	0.99	376.95	4	108	< 0.001

N = 140. Two-way random effects model where both rater effects and measures effects are random, single measures. Type A ICC using an absolute agreement definition

*ICC* Intraclass correlation coefficient, *SIS* Suicide intent scale

Following Koo and Li’s guidelines for interpreting ICCs [15], the RRRS Total indicates excellent interrater reliability, with an ICC of 0.93 (CI95 0.82–0.99), although the confidence interval extends into a lower level. This level of interrater reliability aligns closely with the original estimates for the RRRS [35], which ranged from 0.93 to 0.95. We conducted the same ICC analysis with the SIS Total and found an excellent estimated ICC of 0.94 (CI95 0.83–0.99).

Table 4 shows the ICC estimates for the individual RRRS test items. The ICC ranged between 0.76 and 0.89, indicating good reliability. In our data, the individual SIS items had greater variability, with the ICCs ranging from 0.34 to 0.87, indicating that three items had poor interrater reliability, eight items had moderate interrater reliability, and four items had good interrater reliability. While the overall ratings were similar for the RRRS and SIS, the level of agreement for the individual items was better for the RRRS. The partially poor interrater reliability on the item level indicates that the subscale and total rating levels are the relevant levels of analysis.

**Table 4:** ICC for each item of the RRRS and SIS

		15 SIS items	
		SIS1: Isolation	0.74
10 Risk-rescue items		SIS2: Timing	0.68
		SIS3: Precautions	0.67
		SIS4: Notification	0.87
		SIS5: Final acts	0.43
Risk 1: Agent used	0.89	SIS6: Preparation	0.73
Risk 2: Consciousness	0.79	SIS7: Suicide note	0.34
Risk 3: Lesions/Toxicity	0.76	SIS8: Communication	0.73
Risk 4: Reversibility	0.88	SIS9: Purpose	0.70
Risk 5: Treatment	0.85	SIS10: Expectation	0.87
Rescue 1: Location	0.79	SIS11: Method conception	0.65
Rescue 2: Rescuer	0.85	SIS12: Seriousness	0.84
Rescue 3: Discovery	0.78	SIS13: Death wish	0.84
Rescue 4: Accessibility	0.89	SIS14: Rescuability	0.47
Rescue 5: Delay	0.84	SIS15: Premeditation	0.58

N = 140

*ICC* Intraclass correlation coefficient *RRRS* the risk-rescue rating scale, *SIS* suicide intent scale

### Case rating levels and use of scale range

**Fig. 1:**
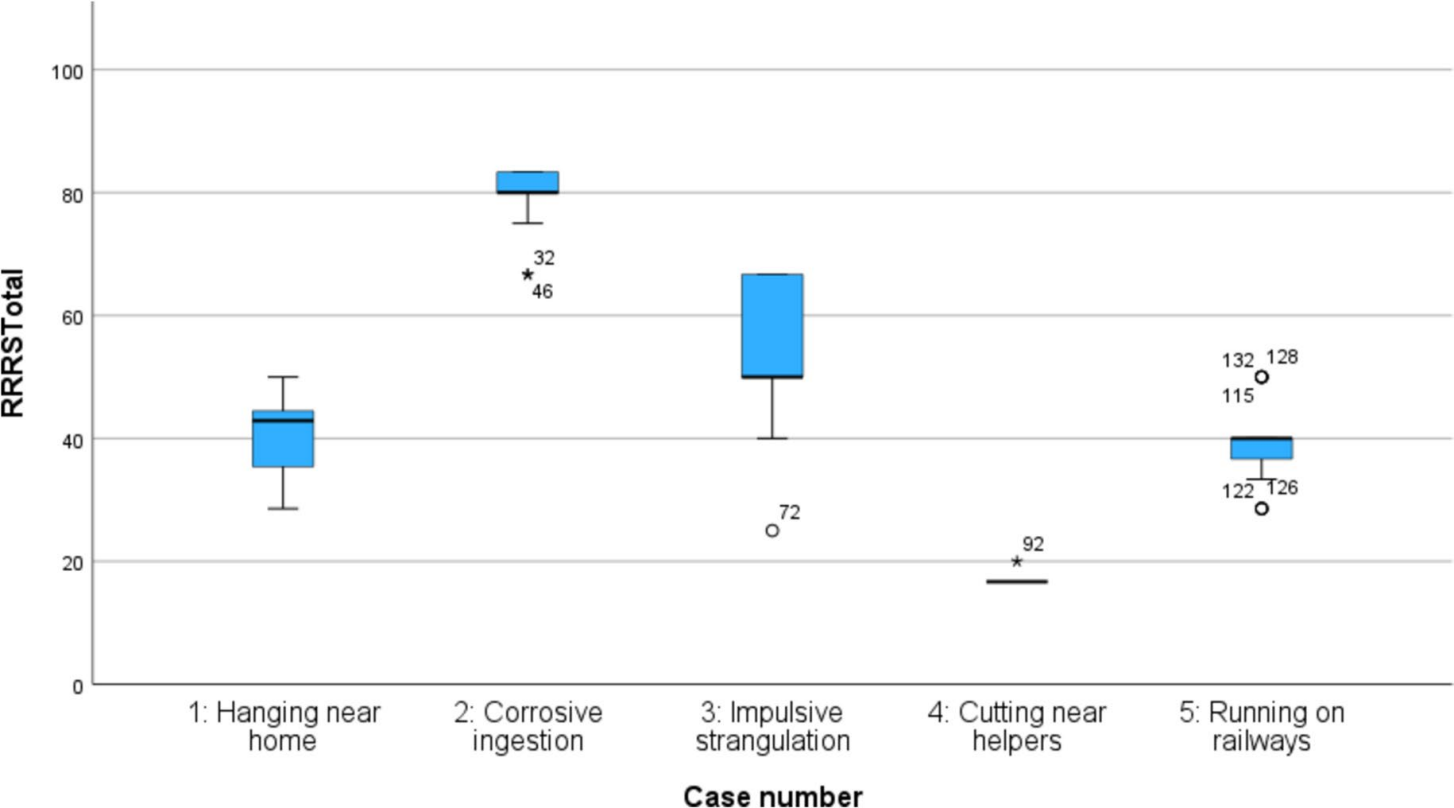
*Boxplot of RRRS total for all five video cases***.** N = 140. *RRRS* the risk-rescue rating scale

We examined whether the clinicians’ ratings of the five video cases corresponded to our expectations of the rating range and levels (as shown in Table 1). Figure 1 graphically presents the clinicians’ ratings of the RRRS Total. The results for the RRRS Total, SIS Total, and RRRS subscale variables compared to our expected rating levels are presented in Table 5.

**Table 5:** Mean ratings and standard deviations for all five video cases and expected rating levels for RRRS total and subscales and SIS total

	RRRS Total (range 16–83)	Risk subscale (range 5–15)	Rescue subscale (range 5–15)	SIS Total (range 0–30)
Case 1 Hanging near home Mean rating (SD) Expected rating	40.4 (6.2) (40–50)	9.4 (1.5) (9–10)	13.2 (0.8) (12–13)	18.2 (2.5) (7–17)
Case 2 Corrosive ingestion Mean rating (SD) Expected rating	80.1 (4.5) (70–83)	12.0 (1.5) (13–15)	5.5 (0.8) (5–7)	22.1 (1.5) (18–30)
Case 3 Impulsive strangulation Mean rating (SD) Expected rating	56.5 (10.8) (50–60)	6.8 (0.9)* (9–10)	6.3 (1.5) (5–7)	10.3 (2.1) (7–17)
Case 4 Cutting near helpers Mean rating (SD) Expected rating	16.8 (0.6) (16–30)	5.0 (0.0) (5–6)	14.6 (0.6) (14–15)	1.4 (1.4) (0–6)
Case 5 Running on railways Mean rating (SD) Expected rating	39.3 (5.9) (30–40)	7.1 (0.3) (7–8)	11.0 (1.4) (10–11)	15.2 (2.8) (7–17)

Case 3, highlighted in bold

N = 140. SD: standard deviation

*RRRS* The risk-rescue rating scale, SIS suicide intent scale. Expected ratings are cited from the study plan presented in Table 1

*Mean rating outside the expected rating range

The ratings for the RRRS Total, SIS Total, and Rescue subscales aligned with our expectations from the study plan outlined in Table 1. One case (case 3) had lower ratings on the Risk subscale than expected, yielding a low rather than moderate rating, although the RRRS Total ratings were as expected also for this case. The ratings varied between the cases, and the raters made use of the entire range of the scale. This indicates that the video cases functioned as intended, displaying a range of levels of lethality and suicide intent.

### Clinical group differences in rating

To examine a possible difference in ratings between teams with different clinical experience and tasks, we recorded information about the clinical role of the participants. Since the RRRS Total ratings differed greatly among the video cases, we tested the influence of group belonging separately for each case. Table 6 shows the ANOVA tests of the influence of different clinical team groupings on each of the RRRS Total ratings.

**Table 6:** ANOVA test of the influence of clinical team grouping on rrrs total rating for all five video cases

	Mean square between groups	Mean square within groups	F	*p*
Case 1: Hanging near home	47.968	37.917	1.265	0.309
Case 2: Corrosive ingestion	30.794	18.588	1.657	0.203
Case 3: Impulsive strangulation	56.547	124.492	0.454	0.717
Case 4: Cutting near helpers	0.238	0.417	0.571	0.639
Case 5: Running on railways	28.382	36.200	0.784	0.515

*RRRS* The risk-rescue rating scale, *SIS* suicide intent scale. Degrees of freedom for each case: df between groups: 3; df within groups: 24

The case ratings revealed no significant differences between the teams.

### Correlations between the RRRS and SIS ratings

We examined the concurrent and discriminant validity of the RRRS by examining the correlations between the RRRS and SIS Total scores. Table 7 presents the results.

**Table 7:** Correlations between RRRS and SIS total ratings and subscales

	RRRS Total	SIS Total	SIS Method	SIS Intent	Risk
SIS Total	0.726^**^				
SIS Method	0.797^**^	0.908^**^			
SIS Intent	0.584^**^	0.948^**^	0.728^**^		
Risk	0.791^**^	0.839^**^	0.770^**^	0.789^**^	
Rescue	− 0.896^**^	− 0.489^**^	− 0.649^**^	− 0.309^**^	− 0.478^**^

N = 140

*RRRS* the risk-rescue rating scale, *SIS* suicide intent scale

**Correlation is significant at the 0.01 level (2-tailed)

The correlation between the RRRS and SIS Totals was high (0.73) and was even greater between the RRRS Total and the SIS Method subscale (0.80) and lower for the SIS Intent subscale (0.58). The lowest correlation (− 0.48) was between the Risk and Rescue subscales, indicating that they tapped distinct, though overlapping, constructs.

## Discussion

Our study indicates that clinicians were able to use the RRRS with an excellent level of interrater reliability. The ratings were independent of clinical position, occupational background, or department. When using ratings on SIS as a benchmark, the RRRS ratings reached a similar level of interrater reliability, which was also similar to the original RRRS estimates [35].

### RRRS and SIS interrater reliability

Although the level of interrater reliability indicated by our study was excellent for both the RRRS and SIS, we should note that this level of interrater agreement was influenced by our study design. This design made use of the full rating range of the instruments, intentionally causing much variation among the five cases. Since the ICC measures variation between cases compared to variation between raters, the ICC is naturally high in such a study design. In contrast, a different study design with a very low level of variation between cases would be expected to result in lower ICC levels. Thus, rather than focusing on absolute measures of interrater reliability, our interest also lay in comparing the performance of the two instruments under equivalent conditions. Here, we observe that while both instruments had excellent overall levels of interrater reliability, the ICC item-level estimates were lower for the SIS, with three of fifteen items recording a poor level. All ten RRRS items had good or excellent ICC level estimates.

Due to the dearth of contemporary research on lethality measures, it is difficult to establish a baseline with which to compare our findings on reliability and validity. A search of PsychInfo yielded no reviews of the psychometric properties of the RRRS. A Korean study of emergency room assessment [14] finds that the RRRS predicts hospitalization after a suicide attempt better than does the SIS and that the combination outperforms each individual measure. Another relevant study [18] did not address interrater reliability, focusing instead on the factor structure of the RRRS, interestingly identifying a three-factor structure. In their French sample of 608 suicide attempters, an additional factor called "Implementation" emerged alongside risk and rescue factors. Our study’s design, with a small number of cases, is unsuitable for factor analysis, leaving the question of factor structure unaddressed.

Spirito et al. [31] expressed objections to the use of the RRRS with adolescent suicide attempters. They argue that adolescents tend to use suicide methods of low lethality, particularly with a high chance of rescue. This can result in a “floor effect”, causing problems for interrater reliability. While acknowledging this problem, we do not see this as a fundamental problem for the present study. Our aim is not to distinguish between finer degrees of low lethality suicide attempts, but rather to find a way to separate serious attempts from less serious ones. Indeed, highlighting the possible differences between adult and adolescent suicide attempts would be useful for the field and for clinicians. Interestingly, Spirito and colleagues’ claim that adolescents tend to use methods of low lethality is hard to confirm empirically without developing the very measuring methods they criticize.

The SIS has been extensively examined, and Freedenthal [8] reported interrater reliability coefficients that align well with our findings.

### Concurrent and discriminant validity of the RRRS compared to self-report methods

A major problem with the use of self-reports in preventing or predicting suicide is that patients are often unwilling or unable to report their intentions [20]. The option to supplement self-reports with brief clinician-rated lethality measures may enhance the validity of such efforts.

In our study, the RRRS and SIS ratings correlated, as anticipated. The correlation was stronger between the RRRS Total and the SIS subscale Methods, which is most similar to the RRRS, and weaker for the SIS subscale Intent, which is less similar to the RRRS. This similarity and contrast to the criterion measure attests to the good concurrent and discriminant validity of the RRRS. The RRRS appears to assess a construct related to, but also distinct from, the patient-reported suicide intent which is assessed in the SIS.

It is clinically valuable to obtain separate measures of the lethality and intent of a suicide attempt, and also to differentiate between self-reports and clinician ratings. These nuances highlight the differences between individuals’ subjective, self-reported perceptions of danger and attempted objective measures of lethality.

Although self-reported expressions of suicide intent might seem like clear indications that a person wishes to die, we know that clinical reality is far less straightforward. It is important to patients to communicate their need for help, and many patients have negative experiences of not feeling understood after self-harm, leading them to express their distress even more forcefully [17]. In this environment, different assessment methods may yield inconsistent responses from participants [5].

A second complication in the use of self-reports when assessing suicidal behavior is that they are typically recorded after the event, sometimes much later. Intense emotions or the influence of intoxicants often make it difficult to recall one’s own experiences or intentions during the event, rendering accurate self-reporting challenging. Gjelsvik and colleagues [10] demonstrated that for a group of patients who had attempted suicide, there was no relation between their self-reported suicidal intent and the lethality of the suicide attempt, when asked three months after the event.

### Lethality measures and patient experience

The final and potentially most significant consideration when employing lethality measures is how clinicians should use the results in meetings with patients. Describing suicide attempts as “low lethality” may inadvertently downplay the personal tragedies and intense emotions experienced by those who have gone through them. Clinicians must exercise great care in how they utilize and comment on lethality assessments. In particular, they must be mindful of the valid fear of criticism and disparagement among patients presenting with suicidal issues in emergency rooms. A common concern among individuals seeking help against suicidal impulses is that clinicians might doubt the severity of their problem, potentially leading them to resort to desperate acts to convince clinicians of their urgent need for help and potentially escalating into more serious suicide attempts [22]. Adolescents also worry that clinicians may focus solely on physical damage from self-harm or on safety measures against physical risk, neglecting to address the underlying problems causing self-harm or suicide attempts [6].

Lethality assessment must not be used for this purpose. The objective of improved assessment is not to dismiss nonlethal suicidality nor to reduce assessment to a mechanical evaluation of risk; our intention is quite the opposite. Employing a brief lethality instrument frees up time, attention, and treatment resources for the highly meaningful work of understanding and helping patients with the problems they need to address.

## Limitations and Further Research

In this study, we opted to use filmed clinical interviews rather than written vignette cases, as this approach is much more realistic for assessing complex dialogue. Filming actual interviews with suicidal adolescents would have been even closer to clinical reality but would have raised difficult concerns regarding ethics and informational safety. Thus, we considered the video role-play approach to be the preferable ethical middle ground between realism and patient exposure.A disadvantage of the role-play interview design is that, in contrast to assessing written vignettes, watching videos is time-consuming. Administering a large number of such cases to a representative sample of clinicians would be beyond the means of our study. Thus, we confined the study to five cases, although a greater number of cases would have been better for the statistical analysis of interrater reliability.

For future research, studies utilizing data from actual patients represent a natural next step. Our current design is limited to a few constructed cases, and it is important to compare this to a wide range of real patients with different degrees of lethality. This would enable us to address Misson’s (2010) finding of a three-factor structure in their RRRS sample. A qualitative study of clinicians’ experiences with the RRRS as a tool for communication within health services would be important, as would a study exploring patients’ experiences of being assessed.

Once we have confidence in the instrument’s psychometric properties, it should prove a useful moderator variable. Many studies treat suicide attempts as a dichotomous variable [7, 27], while the lethality perspective adds a continuum nuance, which may increase our explanatory power. Combined with knowledge about suicide intent, this nuance may make important contributions to understanding and predicting suicidal behavior. Also, lethality measures can provide new knowledge about suicide attempts in different groups and settings, for instance differences between adolescents and adults or the development of lethality in patients with several suicide attempts.

## Conclusion

Our study examined the interrater reliability and discriminant validity of the Risk-Rescue Rating Scale when assessing role-played video cases. We found that clinicians were able to use the instrument at asimilar level of interrater reliability to the well established Suicide Intent Scale. The RRRS may help clinicians communicate better about suicide attempts and improve assessment-based reporting. The instrument should be further tested in a clinical setting involving a broad variety of real patients. The final argument for including lethality measures in routine clinical practice must be whether it actually alleviates our problems of assessment and communication.

## Data Availability

The dataset supporting the conclusions of this article is available upon reasonable request to the corresponding author.
